# Supplementary material for: Activation of nuclear receptors correlates with tuberculosis severity and is a target for host-directed therapy
Source: Front Cell Infect Microbiol. 2025 Dec 16;15:1724798. doi: 10.3389/fcimb.2025.1724798 (PMC12748203; doi:10.3389/fcimb.2025.1724798)
Supplement: Supplementary file 2 [file DataSheet2.pdf]

Supplementary Table 3. List of primer-probes used in this study.

Gene	Primer - probe
Abca1	Mm00442646_m1
Abcg1	Mm00437390_m1
Lpcat3	Mm00520147_m1
Srebf1	Mm00550338_m1
Arg1	Mm00475988_m1
Arg2	Mm00477592_m1
Nr1h3	Mm00443451_m1
Apoe	Mm01307193_g1
Hprt1	Mm03024075_m1
Hmbs	Mm01143545_m1

Supplementary Table 4. List of flow cytometry antibodies used in this study.

Target	Clone	Fluorochrome	Catalogue* #	RRID
CD4	GK1.5	BV786	100453	AB_2565843
CD8	53-6.7	BV650	100741	AB_2563056
CD11b	M1/70	BV510	101245	AB_2561390
CD11c	N418	PE-Cy7	117318	AB_493568
CD19	6D5	APC-Cy7	115530	AB_830707
CD45	30-F11	PE	103106	AB_312971
Ly6C	HK1.4	BV421	128014	AB_1732079
Ly6G	1A8	PercP-Cy5.5	127616	AB_1877271
SiglecF	S17007L	APC	155508	AB_2750237

* all Biolegend
